# Corticosteroid-Induced Myopathy

**DOI:** 10.7759/cureus.49548

**Published:** 2023-11-28

**Authors:** Andreia Coutinho, Núria Fonseca, Isabel Novo, Luciana Faria, Violeta Iglesias

**Affiliations:** 1 Internal Medicine, Centro Hospitalar do Médio Ave, Santo Tirso, PRT

**Keywords:** corticosteroid-induced myopathy, muscle biopsy, acute myopathy, corticosteroid side effects, toxic myopathy

## Abstract

Corticosteroid-induced myopathy is the most common drug-induced myopathy and could appear during the treatment of diseases where corticosteroids are the mainstay of treatment. We present a clinical case of a patient treated with corticosteroids who presented with proximal muscle weakness, myalgias, marked elevation of muscle enzymes, and acute kidney injury due to rhabdomyolysis. The definitive diagnosis was only possible through a muscle biopsy.

## Introduction

Myopathy is an important and frequent complication of the use of various drugs, such as corticosteroids [1]. Chronic myopathy occurs when corticosteroids are used for more than four weeks and in doses exceeding 10 mg of prednisolone per day (or equivalent), whereas acute myopathy is more often associated with critically ill patients and the use of more than 60 mg per day of prednisolone (or equivalent) [1]. One of the most relevant differential diagnoses is polymyositis, an inflammatory myopathy. On clinical evaluation, both are characterized by predominantly proximal muscle weakness (neck flexors and proximal limb muscles), but pain is a rare feature of steroid-induced myopathy [2,3]. Muscle enzymes are normal or slightly elevated in corticosteroid-induced myopathy, unlike polymyositis, and specific myositis autoantibodies are present in up to 85% of cases of inflammatory myopathies [4]. Often, only muscle biopsy and response to treatment distinguish them.

## Case presentation

This is the clinical case of a 75-year-old female with a history of hypertension and cirrhosis due to autoimmune hepatitis/primary biliary cirrhosis overlap syndrome (treated with prednisolone and ursodeoxycholic acid); the latter had resulted in multiple hospitalizations and emergency episodes in the previous six months. She returned to the emergency department due to increased asthenia, generalized painful complaints, and a perception of low urinary output. On physical examination, she exhibited psychomotor retardation, flapping, and skin and mucous membrane dehydration. In an internal medicine consultation one week earlier, she had been advised to discontinue diuretics (spironolactone and furosemide), but the patient continued to take them. Diagnostic tests showed hyperlactacidemia (4 mEq/L), hyponatremia (119 mEq/L), and acute kidney injury (creatinine: 2.4 mg/dL), with hyperkalemia (7.4 mEq/L) and metabolic acidosis (bicarbonate: 16 mEq/L). Echocardiography showed a collapsed inferior vena cava. Diuretics were stopped, and fluid therapy was initiated for hydration and correction of ionic disturbances. The patient improved during the first three days of hospitalization with resolution of these issues. However, while in the hospital, she began to complain of progressively worsening myalgias in the lower limbs and had elevated muscle enzymes at this stage.

Considering the most likely diagnostic hypothesis of inflammatory myopathy, a panel of specific myositis autoantibodies was requested, the patient underwent muscle biopsy, and treatment was initiated: corticosteroid dose was increased to 1 mg/kg/day, and fluid therapy was resumed. Despite initial improvement, which was only transient, the patient eventually worsened in terms of symptoms and muscle weakness, ultimately presenting with proximal predominant tetraparesis with proximal plegia in all four limbs (distal grade 3), increasing muscle enzymes, deteriorating renal function with low urinary output, ionic disturbances, and worsening liver biomarkers.

**Figure 1:**
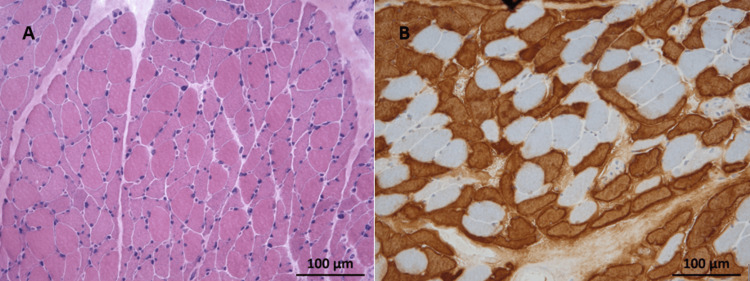
Histology Images of Muscle Biopsy Hematoxylin and eosin staining (A) displays frequent atrophied fibers with predominantly angular contours scattered throughout the fascicles, in the absence of necrotic fibers, basophilic fibers, or inflammatory infiltrates. Immunohistochemistry for fast myosin (B) reveals that most of the atrophied fibers correspond to type 2 fibers.

The results of diagnostic tests and their temporal evolution are shown in Table 1. With the diagnosis of oliguric acute kidney injury due to rhabdomyolysis, unresponsive to medical therapy, transfer to nephrology for renal replacement therapy was proposed and accepted. However, during the preparation for transfer, the patient became hemodynamically unstable, with hypotension even with fluid therapy, and was admitted to the emergency room. Despite the initiation of vasopressor support, the patient experienced cardiorespiratory arrest, and resuscitation was not successful. Only later were we able to access the results of the immunological panel for myositis (all negative) and the muscle biopsy: type 2 fiber atrophy, rare fibers in the process of necrosis, and absence of inflammatory infiltrates, findings consistent with the diagnosis of corticosteroid-induced myopathy (Figure 1) [5].

**Table 1:** Analytical Data Related to the Clinical Presentation of Myalgia ALT: alanine aminotransferase, AST: aspartate aminotransferase, GGT: gamma-glutamyl transferase

Analytical parameters	Initial	Evolution	Reference range
Creatine kinase (U/L)	2,323	3,844	29-168
Myoglobin (ng/mL)	19,844	48,408	14.3-65.8
Creatinine (mg/dL)	0.87	1.57	0.57-1.11
Urea (mg/dL)	98	252	20.9-43
Potassium (mEq/L)	3.9	6.5	3.5-5
Phosphorus (mg/dL)	-	8.95	2.3-4.7
Lactate dehydrogenase (UI/L)	580	1,010	125-248
AST (UI/L)	109	314	5-34
ALT (U/L)	116	542	0-55
Total bilirubin (mg/dL)	1.6	3.17	0.2-1.2
Direct bilirubin (mg/dL)	1.3	2.36	<0.5
GGT (UI/L)	131	85	9-36
Alkaline phosphatase (U/L)	122	138	40-150

## Discussion

The patient presented with acute myopathy, which was initially interpreted in an inflammatory context, given the presence of myalgias (common in inflammatory myopathies, absent in toxic myopathies [6]). Additionally, the patient's autoimmune background, having overlap syndrome of two autoimmune diseases, led to clinical suspicion of autoimmune myositis, with concomitant treatment with immunosuppressive therapy. Autoimmune myositis, when treated correctly, usually has a good prognosis [2]. The initial partial positive response also supported this hypothesis. However, the subsequent worsening of both muscle enzymes and renal function due to significant rhabdomyolysis marked an unfavorable outcome, casting doubt on the diagnosis of inflammatory myopathy.

In this case, the muscle biopsy was essential for the final diagnosis, although it was only available after the patient's death. The histological characteristics of polymyositis are necrosis, degeneration and regeneration of muscle fibers, and infiltration of inflammatory cells [4], which are not compatible with the findings in this patient. On the other hand, histological evaluation in a case of corticosteroid-induced myopathy shows severe atrophy of muscle fibers with a preference for type 2b fibers, and the absence of inflammatory infiltrate [5], precisely the changes found in this patient's biopsy.

The treatment of corticosteroid-induced myopathy is based on discontinuing corticosteroid therapy [5,6], and usually, the prognosis is good, with partial to complete recovery. However, recovery can be slow, extending over six months or beyond [7], and there is a risk of chronic and irreversible changes, even in the case of acute steroid myopathy [8]. In this case, the patient experienced a progressive worsening of rhabdomyolysis and subsequent acute kidney injury, ultimately leading to her death. This was contributed by the delayed diagnosis and the absence of timely discontinuation of corticosteroids.

## Conclusions

This case highlights an atypical presentation of acute corticosteroid-induced myopathy in a patient who had been on corticosteroid therapy for several months and experienced severe and unfavorable progression in just a few days. Steroid-induced myopathy is a diagnosis of exclusion and is sometimes disregarded, leading to delays in establishing the final diagnosis.
